# Changes in HER2, ER, PR, and Ki-67 in HER2-Negative Breast Cancer After Neoadjuvant Chemotherapy: A Case–Control Study

**DOI:** 10.3390/curroncol33010006

**Published:** 2025-12-21

**Authors:** Youzhao Ma, Yan Yang, Mingda Zhu, Yue Yu, Xin Wang

**Affiliations:** 1 The First Department of Breast Cancer, Tianjin Medical University Cancer Institute and Hospital, National Clinical Research Center for Cancer, Huan-Hu-Xi Road, He-Xi District, Tianjin 300060, China; zlyymayouzhao3773@zzu.edu.cn (Y.M.); yuyue@tmu.edu.cn (Y.Y.); 2 Tianjin’s Clinical Research Center for Cancer, Tianjin Medical University, Tianjin 300060, China; 3 Key Laboratory of Breast Cancer Prevention and Therapy, Tianjin Medical University, Ministry of Education, Tianjin 300060, China; 4 Key Laboratory of Cancer Prevention and Therapy, Tianjin Medical University, Tianjin 300060, China; 5 Department of Breast Disease, Henan Breast Cancer Center, The Affiliated Cancer Hospital of Zhengzhou University & Henan Cancer Hospital, No. 127, Dongming Road, Zhengzhou 450000, China; 6 Department of General Surgery, The Second Hospital of Chifeng, Chifeng 024000, China; llyyyangyan@yeah.net; 7 Fudan University Pudong Medical Center, Shanghai Pudong Hospital, 2800 Gongwei Road, Pudong, Shanghai 201399, China; 90259@shpdh.org

**Keywords:** breast cancer, neoadjuvant chemotherapy, HER2 change

## Abstract

**Purpose**: This study investigates receptor status changes following neoadjuvant chemotherapy (NAC) in breast cancer, aiming to identify new therapeutic opportunities and improve human epidermal growth factor receptor 2 (HER2) detection and categorization methods. **Methods**: This retrospective analysis was conducted on patients with breast cancer who underwent NAC and surgery between July 2022 and June 2024. Chi-square tests and logistic regression models were applied to assess the associations between HER2 status changes and clinicopathological features. **Results**: Among 508 patients, the receptor discordance rates after NAC were 5.3% for estrogen receptor (ER), 21.3% for progesterone receptor (PR), and 43.7% for HER2. Ki-67 expression decreased in 64.6% of cases and increased in 6.8%. Of the 103 patients with HER2-0, 47 (45.6%) transitioned to IHC 1+, 9 (8.7%) to IHC 2+/ISH−, and 1 (1.0%) to IHC 2+/ISH+. Among 256 patients with HER2 IHC 1+, 58 (22.7%) transitioned to IHC 2+/ISH−, 36 (14.1%) to IHC 0, and 9 (3.5%) to IHC 2+/ISH+. For 149 patients with HER2 IHC 2+/ISH−, 50 (33.6%) transitioned to IHC 1+, 6 (4.0%) to IHC 2+/ISH+, 5 (3.4%) to IHC 0, and 1 (0.7%) to IHC 3+. Univariate analysis revealed that, when compared to grade III tumors, grade I–II tumors exhibited a higher rate of HER2-0 to HER2-low conversion (66.7% vs. 36.8%, *p* = 0.027). HER2-low to HER2-0 conversion was associated with ER negativity (*p* = 0.028), PR negativity (*p* = 0.021), HER2 IHC 1+ (vs. IHC 2+, *p* = 0.001), and TIL >10% (*p* = 0.049). Multivariate analysis revealed that tumors with HER2 IHC 1+ were more likely to convert to HER2-0 after NAC than those with HER2 IHC 2+ (*p* = 0.020). **Conclusions**: Following NAC, ER gain, PR loss, and Ki-67 reduction were common. HER2 and ER status changes predominantly occurred within adjacent expression intensity levels.

## Simple Summary

To identify new therapeutic opportunities, this study investigates receptor status changes following neoadjuvant chemotherapy in breast cancer. Among 508 patients, the receptor discordance rates after neoadjuvant chemotherapy were 5.3% for estrogen receptor, 21.3% for progesterone receptor, and 43.7% for HER2. Ki-67 expression decreased in 64.6% and increased in 6.8% of all cases. Of the 103 patients with HER2-0, 47 (45.6%) transitioned to IHC 1+, 9 (8.7%) to IHC 2+/ISH−, and 1 (1.0%) to IHC 2+/ISH+. Among 256 patients with HER2 IHC 1+, 58 (22.7%) transitioned to IHC 2+/ISH−, 36 (14.1%) to IHC 0, and 9 (3.5%) to IHC 2+/ISH+. For 149 patients with HER2 IHC 2+/ISH−, 50 (33.6%) transitioned to IHC 1+, 6 (4.0%) to IHC 2+/ISH+, 5 (3.4%) to IHC 0, and 1 (0.7%) to IHC 3+. Tumors with HER2 IHC 1+ were more likely to convert to HER2-0 after neoadjuvant chemotherapy than those with HER2 IHC 2+ (*p* = 0.020). The changes in receptors were common. HER2 and estrogen receptor status changes predominantly occurred within adjacent expression intensity levels. These findings provide insights into the mechanisms underlying estrogen receptor and HER2 transitions.

## 1. Introduction

Breast cancer remains the leading malignancy threatening women’s health worldwide [1,2,3]. Molecular subtype based on estrogen receptor (ER), progesterone receptor (PR), and human epidermal growth factor receptor 2 (HER2) has established the foundation for individualized breast cancer treatment [4]. ER pathway blockade inhibits the proliferation of ER-positive breast cancer, and endocrine therapy has significantly improved the overall patient survival. The discovery of HER2 [5] was a milestone in breast cancer treatment. HER2 expression, as assessed using immunohistochemistry (IHC) and in situ hybridization (ISH), categorizes breast cancer into HER2-positive (HER2 overexpression) and HER2-negative subtypes [6]. HER2-negative includes IHC 0, 1+, and 2+ with no ISH amplification, whereas HER2-positive includes IHC 3+ and IHC 2+ with ISH amplification. The HER2-low category comprises IHC 1+ and IHC 2+/ISH-negative cases. Traditional anti-HER2 therapies, such as trastuzumab and pertuzumab, have significantly improved survival in patients with HER2-positive status [7,8] but have shown limited efficacy in HER2-negative cases [9]. The DESTINY-Breast04 trial demonstrated that the novel antibody-drug conjugate (ADC), trastuzumab deruxtecan, exhibits significant therapeutic efficacy in HER2-low advanced breast cancer [10], likely due to its unique bystander effect [11]. Although most studies do not support HER2-low breast cancer as a distinct biological subtype [12,13,14], it is increasingly recognized as a therapeutic category.

However, HER2 status is unstable, and a proportion of patients experience changes in HER2-0 and HER2-low expression following neoadjuvant therapy (NAT) or recurrence [15,16,17,18]. This variability can largely be attributed to HER2 heterogeneity, including both temporal and spatial differences in the expression [19,20,21,22]. Due to sampling limitations, HER2 assessment in biopsy specimens may not fully represent tumor heterogeneity, causing discrepancies in HER2 status post-NAT. In addition, substantial interobserver variability exists in pathologists’ interpretation of HER2 IHC 0 and 1+ [23,24], thereby further contributing to inconsistencies in HER2 expression following NAT. A detailed understanding of these changes is critical for refining HER2 detection and interpretation standards. The expression of Ki-67 after neoadjuvant endocrine therapy (NET) is associated with prognosis [25]. Ki-67 is also a prognostic factor for patients with breast cancer receiving neoadjuvant chemotherapy (NAC) [26]. Changes in Ki-67 after NAC are associated with the prognosis of breast cancer [27], and ER and PR status may be altered post-NAC [28]. These receptor modifications are of particular interest as they may reflect tumor escape mechanisms and provide new therapeutic opportunities. For instance, tumors transitioning from HER2-0 to HER2-low may become candidates for novel ADC therapies, whereas patients whose ER or PR status shifts from negative to positive may benefit from endocrine therapy.

Investigating receptor changes after NAT can help identify novel treatment strategies and improve HER2 detection and categorization. However, limited studies have specifically examined HER2 IHC 0, 1+, and 2+ changes post-NAT. Notably, HER2 alterations occur after NAC rather than after NET [18]. This study aimed to analyze post-NAC changes in HER2, ER, PR, and Ki-67 by evaluating data from our center, providing valuable clinical insights into receptor dynamics, and laying the groundwork for further research.

## 2. Materials and Methods

### 2.1. Patient Selection

The clinicopathological data of patients with breast cancer who underwent surgery at the First Department of Breast Cancer, Tianjin Medical University Cancer Institute and Hospital, from 1 July 2022 to 30 June 2024, after receiving NAC, were collected. The inclusion criteria were female gender, age ≥ 18 years at the time of diagnosis, stages I–III invasive breast cancer, HER2-negative, received 4–8 cycles of standard NAC followed by radical surgery, not reaching pathological complete response (pCR), with available ER, PR, and HER2 results of biopsy specimen, and residual tumor, and HER2-negative before NAC. The exclusion criteria included stage IV, bilateral breast cancer, participation in clinical trials of new drugs, having received inhibitors of immune checkpoints in the stage of NAT, having received NET, non-pCR but residual tumors not available for IHC and ISH testing, or the presence of other primary malignant tumors.

### 2.2. Data Collection

The following clinicopathological data were collected from clinical medical records: age at diagnosis, menopausal status, height, weight, NAC regimen, histological grading of biopsy specimens, tumor stromal infiltrating lymphocytes (TIL), pre-NAC ER, PR, HER2, Ki-67, baseline clinical T and N staging, and postoperative residual tumor ER, PR, HER2, and Ki-67. The staging was performed in accordance with the American Joint Committee on Cancer Breast Cancer Staging System (version 7). Clinical staging was used before NAC, and pathological staging was applied after NAC. ER/PR < 1% was considered negative, and ER/PR ≥1% was considered positive. ER positivity was categorized into two: 1–50% indicating weakly positive and 51–100% indicating strongly positive. The interpretation of HER2 conformed to the American Society of Clinical Oncology/College of American Pathologists HER2 test guideline. HER2 IHC 0, 1+, and 2+/ISH− were considered HER2-negative, while HER2 IHC 3+ and 2+/ISH+ were considered HER2-positive. HER2-low was defined as IHC 1+ or 2+/ISH−. HER2-0 was defined as HER2 IHC 0. Ki-67 was categorized according to a cut-off value of 30%, with ≤30% defined as a low expression and >30% defined as a high expression. The changes in HER2/ER/PR/Ki-67 after NAC were compared, and the clinicopathological factors associated with HER2 changes were analyzed.

### 2.3. Statistical Analysis

This study used SPSS 23.0 software, R Version 4.2.2, and Adobe Illustrator Version 2023 for statistical analysis. The median values of continuous variables, including age at diagnosis/ER/PR/Ki-67, were calculated through descriptive statistical analysis. The difference in the continuous variables of ER/PR/Ki-67 before and after NAC was analyzed using a paired *t*-test. The difference in the categorical variables of ER/PR/Ki-67 before and after NAC was analyzed using the Chi-square test. The Chi-square test was applied to analyze the association between HER2 changes and clinicopathological features. Variables with *p* < 0.1 in univariate analysis were included in the multivariate regression model for correction. The logistic regression model was employed to analyze the independent factors associated with HER2-low conversion to HER2-0. A bilateral *p* < 0.05 was considered to indicate statistical significance.

## 3. Results

### 3.1. Clinicopathological Characteristics at Baseline and After NAC

A total of 508 patients were included in this study. The majority (98.0%, n = 498) received anthracycline- and taxane-based NAC regimens; hence, the chemotherapy regimens were not included in the analysis. The median age at diagnosis was 49 years (range: 24–75 years). Among the patients, 311 (61.2%) were premenopausal, and 322 (63.4%) had a body mass index (BMI) of ≥24.0. Histological grading was available for 383 cases, with 258 (50.8%) categorized as grade I–II and 125 (24.6%) as grade III. Regarding clinical staging, 37 (7.3%) patients were stage T0–T1, 345 (67.9%) were T2, and 126 (24.8%) were T3–T4. Nodal involvement was as follows: 82 (16.1%) N0, 302 (59.4%) N1, 76 (15.0%) N2, and 48 (9.5%) N3. The median ER expression was 80% (range: 0–90%), with 434 (85.4%) ER-positive patients. Median PR expression was 30% (range: 0–90%), with 364 (71.7%) PR-positive cases. HER2 IHC status was distributed as follows: 103 (20.3%) HER2 IHC 0, 256 (50.4%) HER2 IHC 1+, and 149 (29.3%) HER2 IHC 2+/ISH−, with HER2-low breast cancer accounting for 79.7% of cases. The median Ki-67 index was 35% (range: 3–90%), and 234 patients (46.1%) had low Ki-67 expression (Table 1).

After NAC, tumor (T) and nodal (N) staging showed an overall downward trend, except for an increase in the proportion of stage N3 cases. ER positivity decreased to 419 (82.5%), with a median ER expression of 90% (range: 0–95%). PR positivity declined to 300 (59.1%), with a median PR expression of 5% (range: 0–90%). The median Ki-67 index was reduced to 15% (range: 0–85%), and the number of patients with low Ki-67 expression increased to 395 (77.8%). Post-NAC HER2 status was as follows: 87 (17.1%) HER2 IHC 0, 250 (49.2%) HER2 IHC 1+, 154 (30.3%) HER2 IHC 2+/ISH−, 16 (3.1%) HER2 IHC 2+/ISH+, and 1 (0.2%) HER2 IHC 3+ (Table 1).

### 3.2. Changes in ER, PR, HER2, and Ki-67 After NAC

**Figure 1:**
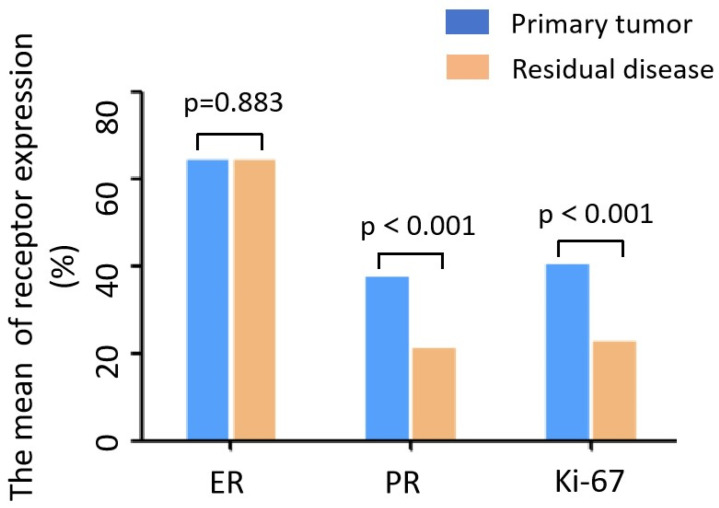
Changes in mean ER, PR, and Ki-67 expression after NAC. ER, estrogen receptor; PR, progesterone receptor.

The mean expressions of ER, PR, and Ki-67 before NAC were 64.2%, 37.5%, and 35%, respectively, and after NAC were 64.3%, 21.2%, and 15%, respectively. The change in the mean ER expression was not significant (*p* = 0.883), whereas the PR expression was significantly downregulated (*p* < 0.001), and Ki-67 expression was also significantly reduced (*p* < 0.001) (Table 2A and Figure 1). The post-NAC discordance rates were 5.3% for ER and 21.3% for PR. ER status changed from positive to negative in 4.8% of cases and from negative to positive in 8.1%. PR loss occurred in 23.6% of cases, while PR gain was observed in 15.3%. The Ki-67 levels decreased in 64.6% of cases and increased in 6.8% (Table 2B).

**Figure 2:**
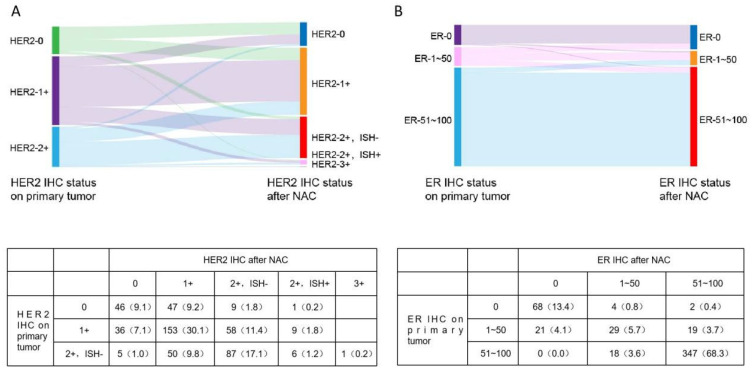
Changes in HER2 and ER after NAC. (**A**) HER2 changes based on IHC 0, 1+, and 2+ category; (**B**) ER changes based on IHC 0, 1–50%, and 51–100% category. ER, estrogen receptor; HER2, human epidermal growth factor receptor 2; IHC, immunohistochemistry; NAC, neoadjuvant chemotherapy.

HER2 IHC/ISH expression showed a discordance rate of 43.7% after NAC. Among patients with HER2 IHC 0, 46 remained unchanged, while 47, 9, and 1 transitioned to IHC 1+, IHC 2+/ISH−, and IHC 2+/ISH+, respectively, showing a gradually decreasing trend. Among those with HER2 IHC 1+, 153 remained unchanged, whereas 58, 36, and 9 transitioned to IHC 2+/ISH−, IHC 0, and IHC 2+/ISH+, respectively. Notably, HER2 IHC 0 and 1+ did not transition to IHC 3+. In patients with HER2 IHC 2+/ISH−, 87 remained unchanged, while 50, 6, 5, and 1 transitioned to IHC 1+, IHC 2+/ISH+, IHC 0, and IHC 3+, respectively (Figure 2A). Among ER-negative patients, 68 remained unchanged, while 4 became weakly positive and 2 became strongly positive. Among those initially weakly ER-positive, 29 remained unchanged, 21 converted to negative, and 19 became strongly positive. In ER strongly positive cases, 347 remained unchanged, 18 became weakly positive, and none transitioned to ER-negative (Figure 2B).

### 3.3. Factors Associated with Changes Between HER2-0 and HER2-Low

Univariate analysis revealed that patients with histological grade I–II had a higher likelihood of transitioning from HER2-0 to HER2-low when compared to those with grade III (66.7% vs. 36.8%, *p* = 0.027). However, age at the time of diagnosis, menopausal status, BMI, TN stage, ER, PR, Ki-67, and TIL were not associated with HER2-0 transformation. Factors significantly associated with the transition from HER2-low to HER2-0 included ER negativity (*p* = 0.028), PR negativity (*p* = 0.021), HER2 IHC 1+ (vs. IHC 2+, *p* = 0.001), and TIL > 10% (*p* = 0.049). No significant associations were detected with age at the time of diagnosis (*p* = 0.071), menopausal status (*p* = 0.072), BMI, histological grade, TN stage, or Ki-67 (Table 3).

Multivariate analysis included variables with *p* < 0.1 from the univariate analysis (age at diagnosis, menopausal status, ER, PR, HER2 status, and TIL). HER2 status emerged as an independent factor influencing HER2-low transition. Tumors with HER2 IHC 1+ were more likely to convert to IHC 0 after NAC compared to those with HER2 IHC 2+ (*p* = 0.020). However, age at the time of diagnosis (*p* = 0.952), menopausal status (*p* = 0.743), ER (*p* = 0.803), PR (*p* = 0.347), and TIL (*p* = 0.085) were not significantly associated with HER2-low to HER2-0 transition (Table 4).

## 4. Discussion

This study provides a detailed analysis of receptor changes, particularly HER2 IHC 0, 1+, and 2+, following NAC. By identifying factors influencing HER2 transitions, these findings offer insights into the potential impact and mechanisms of receptor changes, especially HER2 alterations, on prognosis. In the 508 patients with HER2-negative breast cancer, the ER expression remained relatively stable, PR loss was common, HER2 status frequently changed, and Ki-67 levels generally declined. Univariate analysis revealed that lower histological grades were associated with the transition from HER2-0 to HER2-low, whereas the transition from HER2-low to HER2-0 was associated with ER negativity, PR negativity, HER2 IHC 1+ (vs. IHC 2+), and TIL > 10%. Multivariate analysis identified HER2 IHC 1+ as the only independent factor for HER2-low to HER2-0 conversion.

Past studies have documented ER/PR changes following NAC [28,29,30,31]. Consistent with a previous large-scale retrospective study [30], we noted that ER gains outnumbered ER losses, though the overall mean ER expression remained nearly unchanged. This stability may be attributed to the predominance of weakly positive ER gains. Specifically, among six patients with ER gain, four showed weakly positive and two strongly positive expressions. All 21 patients with ER loss transitioned from weakly positive to negative, and no strongly positive cases directly converted to ER-negative status. In addition, 18 strongly positive ER cases shifted to weakly positive, whereas 19 weakly positive cases converted to strongly positive after NAC. Thus, the ER expression remained relatively stable, with changes occurring primarily between adjacent expression intensities. In contrast, the PR expression was more variable, with a significant reduction in both the median and mean PR levels after NAC. PR loss was notably more frequent than PR gain. The variability of PR expression was also reported by previous studies [28,31]. Currently, it is unclear how the significant loss of PR expression affects prognosis.

Past studies have shown that transitions between HER2-0 and HER2-low expression following NAC are common [15,16,17,18,32,33,34,35]. This study analyzed HER2 transformation based on IHC 0, 1+, and 2+ categories and found that HER2 transitions followed a pattern similar to that of ER, predominantly shifting between adjacent expression intensities. Specifically, HER2-0 transitioned primarily to HER2 IHC 1+, with fewer cases progressing to IHC 2+. Similarly, HER2 IHC 1+ frequently transitioned to either IHC 2+/ISH− or IHC 0, whereas HER2 IHC 2+/ISH− mostly converted to IHC 1+, with occasional direct transitions to IHC 0. Overall, HER2-negative to HER2-positive conversion predominantly occurred via HER2-low, whereas the direct transition from HER2-0 to HER2-positive was rare, a finding consistent with previous reports [33,34]. This suggests that HER2 transition follows an ordered pattern. The transformation of HER2 may reflect both the biological regulation of chemotherapy and HER2 expression heterogeneity. The number of tumor cells declined following NAC; however, the residual tumor cells may represent a population that has developed chemotherapy tolerance. Considering this point, residual tumors from HER2 IHC 2+/ISH− and HER2-0 cases should be reassessed post-NAC to explore potential eligibility for trastuzumab or novel ADC-targeted therapies. In contrast, transitions from either HER2-0 or HER2-low to HER2-positive were relatively infrequent, which is consistent with the findings of Chen et al. [35]. Patients who convert to HER2-positive status after NAC may become eligible for trastuzumab-based targeted therapy, while those who convert to HER2-low expression may be candidates for clinical trials evaluating novel anti-HER2 therapy ADCs.

Ki-67 transition was associated with prognosis [27,36], although one study reported that only post-NAC reductions in Ki-67 in HER2-low breast cancer were prognostically significant [37]. In this study, the median and mean Ki-67 levels significantly declined after NAC. Notably, 64.6% of patients exhibited a shift from high to low Ki-67 expression, whereas only 6.8% transitioned from low to high expression. Most patients showed reduced Ki-67 levels, likely influenced by undergoing 4–6 cycles of NAC. Previous studies have indicated that increased Ki-67 expression post-NAC was associated with poor prognosis [27,36]. However, due to variations in the use of reagent kits and interpretation methods, Ki-67 detection values remain inconsistent, necessitating further research on its post-NAC changes and prognostic implications.

This study has several limitations. As a retrospective study, selection bias is feasible. Patients lacking postoperative receptor data or those enrolled in clinical trials of novel drugs were excluded, which may have affected the observed proportion of HER2-low cases. In addition, the data originated from a single center, and all ER/PR/HER2/Ki-67 assessments were based on initial pathological reports without central laboratory review, representing a key limitation. Furthermore, the study lacks follow-up and survival analysis, warranting further investigation in the future.

## 5. Conclusions

Following NAC, ER, PR, HER2, and Ki-67 exhibited varying degrees of transition. ER changes were minimal and primarily involved shifts to adjacent expression intensity levels. PR loss was more pronounced than PR gain. HER2 changes predominantly occurred between HER2-0 and HER2-low, with transitions largely following adjacent IHC expressions (0, 1+, 2+/ISH−, 2+/ISH+, and 3+). HER2-0 exhibited a higher transition rate than HER2-low. Ki-67 expression was predominantly downregulated. Thus, chemotherapy tends to reduce tumor activity and can subtly shift the biomarker expression, with PR and HER2 being more dynamic than ER. These findings provide insight into tumor adaptation and treatment response rather than random measurement variation.

## Figures and Tables

**Table 1:** Clinicopathological characteristics of primary tumor and residual disease.

Characteristics	Primary Tumor		Residual Disease After NAC	
		N (%)		N (%)
Age at diagnosis (years)		49 (24–75)		
	≤50	281 (55.3)		
	>50	227 (46.7)		
Menopausal status				
	Premenopausal	311 (61.2)		
	Postmenopausal	197 (38.8)		
BMI				
	<24.0	186 (36.6)		
	≥24.0	322 (63.4)		
Histological grade				
	I	4 (0.8)		
	II	254 (50.0)		
	III	125 (24.6)		
	Unknown	125 (24.6)		
T stage				
	T0~Tis	3 (0.6)	T0~Tis	12 (2.4)
	T1	34 (6.7)	T1	217 (42.7)
	T2	345 (67.9)	T2	229 (45.1)
	T3	99 (19.5)	T3	45 (8.8)
	T4	27 (5.3)	T4	5 (1.0)
N stage				
	N0	82 (16.1)	N0	178 (35.0)
	N1	302 (59.4)	N1	166 (32.7)
	N2	76 (15.0)	N2	80 (15.7)
	N3	48 (9.5)	N3	84 (16.5)
ER median		80 (0–90)		90 (0–95)
ER status				
	<1%	74 (14.6)	<1%	89 (17.5)
	≥1%	434 (85.4)	≥1%	419 (82.5)
PR median		30 (0–90)		5 (0–90)
PR status				
	<1%	144 (28.3)	<1%	208 (40.9)
	≥1%	364 (71.7)	≥1%	300 (59.1)
HER2 status				
	0	103 (20.3)	0	87 (17.1)
	1+	256 (50.4)	1+	250 (49.2)
	2+/ISH−	149 (29.3)	2+/ISH−	154 (30.3)
			2+/ISH+	16 (3.1)
			3+	1 (0.2)
Ki-67 median		35 (3–90)		15 (0–85)
Ki-67				
	≤30%	234 (46.1)	≤30%	395 (77.8)
	>30%	274 (53.9)	>30%	113 (22.2)

BMI, body mass index; ER, estrogen receptor; HER2, human epidermal growth factor receptor 2; NAC, neoadjuvant chemotherapy; PR, progesterone receptor.

**Table 2:** Differences in ER, PR, and Ki-67 expression before and after NAC.

**A. Differences Based on the Mean.**			
	**Primary Tumor**	**Residual Disease After NAC**	***p***
ER mean	64.2 (0–90)	64.3 (0–95)	0.883 ^a^
PR mean	37.5 (0–90)	21.2 (0–90)	<0.001 ^a^
Ki-67 mean	40.2 (3–90)	22.5 (0–85)	<0.001 ^a^
**B. Differences Based on IHC Scores.**			
**Primary Tumor**	**Residual Disease After NAC**		***p***
	**Negative/Low N (%)**	**Positive/High N (%)**	
ER status			<0.001 ^b^
<1%	68 (91.9)	6 (8.1)	
≥1%	21 (4.8)	413 (95.2)	
PR status			<0.001 ^b^
<1%	122 (84.7)	22 (15.3)	
≥1%	86 (23.6)	278 (76.4)	
Ki-67 status			<0.001 ^b^
≤30%	218 (93.2)	16 (6.8)	
>30%	177 (64.6)	97 (35.4)	

ER, estrogen receptor; IHC, immunohistochemistry; NAC, neoadjuvant chemotherapy; PR, progesterone receptor. ^a^, Paired *t*-test; ^b^, Chi-square test.

**Table 3:** Univariate analysis of factors associated with HER2-0 and HER2-low transition.

Characteristics	HER2-0 Before NAC (N = 102)			HER2-Low Before NAC (N = 389)		
	Constant 0 N (%)	0 to Low N (%)	*p*	Constant Low N (%)	Low to 0 N (%)	*p*
Age at diagnosis (years)			0.485			0.071
≤50	27 (48.2)	29 (51.8)		186 (86.9)	28 (13.1)	
>50	19 (41.3)	27 (58.7)		162 (92.6)	13 (7.4)	
Menopausal status			0.170			0.072
Premenopausal	26 (40.0)	39 (60.0)		204 (87.2)	30 (12.8)	
Postmenopausal	20 (54.1)	17 (45.9)		144 (92.9)	11 (7.1)	
BMI			0.750			0.687
<24.0	17 (47.2)	19 (52.8)		130 (90.3)	14 (9.7)	
≥24.0	29 (43.9)	37 (56.1)		218 (89.0)	27 (11.0)	
Histological grade			0.027			0.245
I + II	15 (33.3)	30 (66.7)		189 (92.2)	16 (7.8)	
III	12 (63.2)	7 (36.8)		89 (88.1)	12 (11.9)	
T stage			0.247			0.472
T0/Tis/T1	6 (54.5)	5 (45.5)		23 (88.5)	3 (11.5)	
T2	28 (42.4)	38 (57.6)		235 (88.0)	32 (12.0)	
T3	6 (35.3)	11 (64.7)		72 (93.5)	5 (6.5)	
T4	6 (75.0)	2 (25.0)		18 (94.7)	1 (5.3)	
N stage			0.773			0.704
N0	7 (58.3)	5 (41.7)		63 (92.6)	5 (7.4)	
N1	29 (44.6)	36 (55.4)		200 (88.1)	27 (11.9)	
N2	6 (40.0)	9 (60.0)		51 (89.5)	6 (10.5)	
N3	4 (40.0)	6 (60.0)		34 (91.9)	3 (8.1)	
ER status			0.203			0.028
<1%	15 (55.6)	12 (44.4)		36 (80.0)	9 (20.0)	
≥1%	31 (41.3)	44 (58.7)		312 (90.7)	32 (9.3)	
PR status			0.129			0.021
<1%	25 (53.2)	22 (46.8)		79 (83.2)	16 (16.8)	
≥1%	21 (38.2)	34 (61.8)		269 (91.5)	25 (8.5)	
HER2 IHC score						0.001
0						
1+				211 (85.4)	36 (14.6)	
2+/ISH−				137 (96.5)	5 (3.5)	
Ki-67			0.455			0.195
≤30%	14 (40.0)	21 (60.0)		173 (91.5)	16 (8.5)	
>30%	32 (47.8)	35 (52.2)		175 (87.5)	25 (12.5)	
TIL			0.445			0.049
≤10%	28 (42.4)	38 (57.6)		251 (91.6)	23 (8.4)	
>10%	3 (60.0)	2 (40.0)		37 (82.2)	8 (17.8)	

BMI, body mass index; ER, estrogen receptor; HER2, human epidermal growth factor receptor 2; IHC, immunohistochemistry; ISH, in situ hybridization; NAC, neoadjuvant chemotherapy; PR, progesterone receptor; TIL, tumor stromal infiltrating lymphocytes.

**Table 4:** Multivariate analysis of factors associated with HER2-0 and HER2-low transition.

Characteristics	Multivariate Analysis		
	HR	95% CI	*p*
Age at diagnosis (years)			
>50 (versus ≤ 50)	0.949	0.205–4.423	0.952
Menopausal status			
Postmenopausal (versus Premenopausal)	0.762	0.150–3.871	0.743
ER status			
≥1% (versus < 1%)	0.847	0.230–3.121	0.803
PR status			
≥1% (versus < 1%)	0.613	0.221–1.702	0.347
HER2 status			
IHC 2+/ISH− (versus IHC 1+)	0.303	0.110–0.829	0.020
TIL			
>10% (versus ≤ 10%)	2.241	0.894–5.614	0.085

ER, estrogen receptor; HER2, human epidermal growth factor receptor 2; IHC, immunohistochemistry; ISH, in situ hybridization; PR, progesterone receptor; TIL, tumor stromal infiltrating lymphocytes.

## Data Availability

The data presented in this study are available from the corresponding author on reasonable request. The data are not publicly available due to ongoing studies and for patient privacy.

## Abbreviations

ADC	antibody-drug conjugate
BMI	body mass index
ER	estrogen receptor
ISH	in situ hybridization
HER2	human epidermal growth factor receptor 2
IHC	immunohistochemistry
NAC	neoadjuvant chemotherapy
NAT	neoadjuvant therapy
NET	neoadjuvant endocrine therapy
pCR	pathological complete response
PR	progesterone receptor
TIL	tumor stromal infiltrating lymphocytes
